# Maternal Cocaine Administration in Mice Alters DNA Methylation and Gene Expression in Hippocampal Neurons of Neonatal and Prepubertal Offspring

**DOI:** 10.1371/journal.pone.0001919

**Published:** 2008-04-02

**Authors:** Svetlana I. Novikova, Fang He, Jie Bai, Nicholas J. Cutrufello, Michael S. Lidow, Ashiwel S. Undieh

**Affiliations:** 1 Laboratory of Neurogenomics and Proteomics, Department of Biomedical Sciences, University of Maryland, Baltimore, Maryland, United States of America; 2 Laboratory of Integrative Neuropharmacology, Department of Pharmaceutical Sciences, Thomas Jefferson University School of Pharmacy, Philadelphia, Pennsylvania, United States of America; Queensland Institute of Medical Research, Australia

## Abstract

Previous studies documented significant behavioral changes in the offspring of cocaine-exposed mothers. We now explore the hypothesis that maternal cocaine exposure could alter the fetal epigenetic machinery sufficiently to cause lasting neurochemical and functional changes in the offspring. Pregnant CD1 mice were administered either saline or 20 mg/kg cocaine twice daily on gestational days 8–19. Male pups from each of ten litters of the cocaine and control groups were analyzed at 3 (P3) or 30 (P30) days postnatum. Global DNA methylation, methylated DNA immunoprecipitation followed by CGI^2^ microarray profiling and bisulfite sequencing, as well as quantitative real-time RT-PCR gene expression analysis, were evaluated in hippocampal pyramidal neurons excised by laser capture microdissection. Following maternal cocaine exposure, global DNA methylation was significantly decreased at P3 and increased at P30. Among the 492 CGIs whose methylation was significantly altered by cocaine at P3, 34% were hypermethylated while 66% were hypomethylated. Several of these CGIs contained promoter regions for genes implicated in crucial cellular functions. Endogenous expression of selected genes linked to the abnormally methylated CGIs was correspondingly decreased or increased by as much as 4–19-fold. By P30, some of the cocaine-associated effects at P3 endured, reversed to opposite directions, or disappeared. Further, additional sets of abnormally methylated targets emerged at P30 that were not observed at P3. Taken together, these observations indicate that maternal cocaine exposure during the second and third trimesters of gestation could produce potentially profound structural and functional modifications in the epigenomic programs of neonatal and prepubertal mice.

## Introduction

DNA methylation is a major mechanism for the maintenance of epigenetic states [1], [2]. Multiple studies have demonstrated that DNA methylation, generally occurring preferentially at cytosine residues, promotes chromatin repression which inhibits transcription, whereas the absence of methylation is associated with the formation of a chromatin state that is more permissive for transcriptional activity [3]–[9]. DNA methylation has also been implicated in mechanisms that increase genomic stability [8], [10], regulate the expression of parentally imprinted genes [11]–[13], or provide for X chromosome gene dosage compensation in females [14]–[17]. Deficits in DNA methylation in transgenic mice lacking either maintenance DNMT1 (DNA methyltransferase1) or *de novo* DNMT3a, DNMT3b result in lethality at specific stages of development [18], [19]. Perhaps most intriguing is the notion that DNA methylation may contribute to the regulation of developmental and adult gene expression or even that the phenomenon may be capable of providing a memory mechanism for developmentally established gene transcription levels [4], [5], [7], [20]–[24].

Most of the methylated cytosines in mammalian genomes are associated with CpG dinucleotides [25], although non-CpG cytosines could also be methylated [26]. In most genomes, CpGs are relatively under-represented and are found approximately once per 80 dinucleotides. In 1–2% of the genome, however, CpGs form so-called CGIs (CpG islands), which are regions of DNA ranging in size from 200 bp to several kilobases that display high C+G content of >55% and increased CpG frequency with an observed/expected ratio of >0.6 [27]. The mouse genome, for instance, contains ∼37,000 CGIs [28], [29]. A large number of CGIs is associated with the promoters of housekeeping as well as tissue-specific genes [28]. It has been speculated that these CGIs represent memory “footprints” of embryonic gene replication that could influence future gene activation [30]. The possibility that neuroactive xenobiotics such as drugs of abuse could influence or disrupt the memory function of these “footprints” as part of their neurotoxic mechanisms should merit thorough investigation. The present study explored this idea using cocaine as a test agent.

Employing a mouse model of prenatal cocaine exposure, we examined the ability of maternal cocaine exposure to affect DNA methylation in hippocampal pyramidal cells of the offspring. Hippocampal neurons were chosen for this study because cocaine-induced neurochemical, morphological, and physiological alterations in this brain structure have been well documented [31]–[35]. We examined whether chronic maternal cocaine treatment resulted in changes in global DNA methylation or expression of DNMTs in offspring pyramidal neurons on postnatal day 3 (P3). CGIs in the DNA of hippocampal pyramidal neurons were profiled for drug-induced alteration in their methylation state, followed by detailed analysis of methylated CpGs in ten selected gene promoters. The stability of cocaine-related changes at P3 was investigated by re-examining the methylation indices in P30 littermates of the assessed neonates. The findings indicate a strong association between maternal cocaine exposure and significant alterations in global DNA methylation, in CGI-specific methylation, and in the transcriptional activities of several genes some of which are known to code for proteins involved in critical neural functions.

## Results

### Cytoarchitecture, Microdissection, and DNA Content of Hippocampal Pyramidal Layers

**Figure 1:**
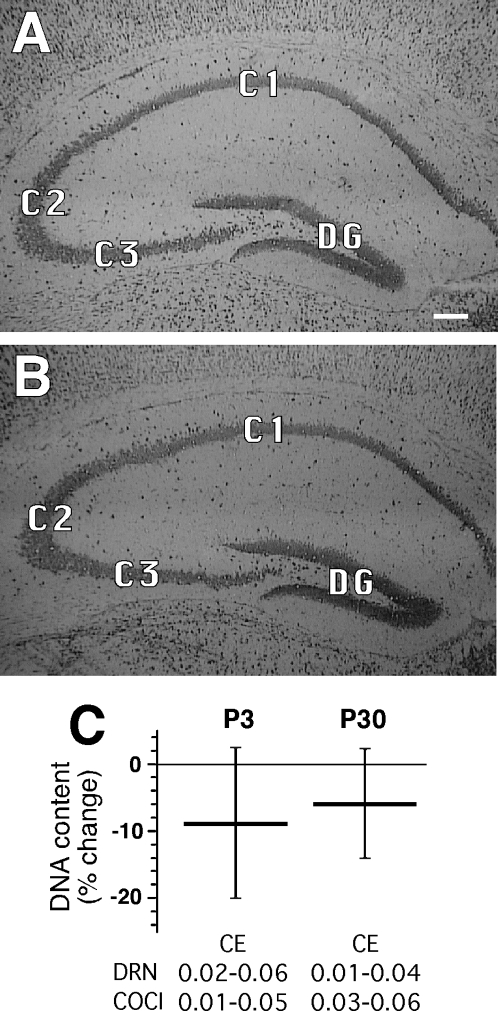
Evaluation of the structure and total DNA content of hippocampal pyramidal tissues in P3 progeny of saline-treated and cocaine-treated mice. (a) Typical micrograph of HistoGene-stained coronal section through the hippocampus of a P3 Saline pup. (b) Micrograph of a comparable section from an age-matched offspring of a cocaine-treated mother. (c) Relative amounts of total DNA content between the offspring of saline-treated and cocaine-treated mothers at P3 and P30. Horizontal bars show mean difference in DNA content between Cocaine and Saline groups at each age, while vertical bars show the SEM (n  = 10/group/age). For either age group, the inter-sample ranges of coefficient of error (CE) values are provided at the bottom of the chart. C1, C2, C3, hippocampal regions; DG, dentate gyrus. Note the absence of detectable histological differences between micrographs a and b.

Qualitative visual analysis of images of coronal histological sections through the hippocampus of the P3 and P30 male offspring of saline-control and cocaine-treatment mothers revealed no inter-group differences at either age. The shape of the entire hippocampal structure was similar between the control and treatment groups on all sections cut at comparable levels. Pyramidal layers had sharp borders without cellular dysplasia. The striata oriens and radiatum showed no abnormal cellular densities or unusual cellular aggregations (Fig. 1a, b).

At both P3 and P30, the estimated total DNA content of the hippocampal pyramidal layer showed no significant differences between the male offspring of saline-control and cocaine-treated mothers (Fig. 1c). Overall, the DNA content of the hippocampal pyramidal layer at both ages ranged from 9.6 to 13.7 µg. The intra-specimen coefficient of error values for the measured DNA content of all the examined hippocampal pyramidal layers were below 0.06 (Fig. 1c), indicating acceptable levels of precision [36].

**Figure 2:**
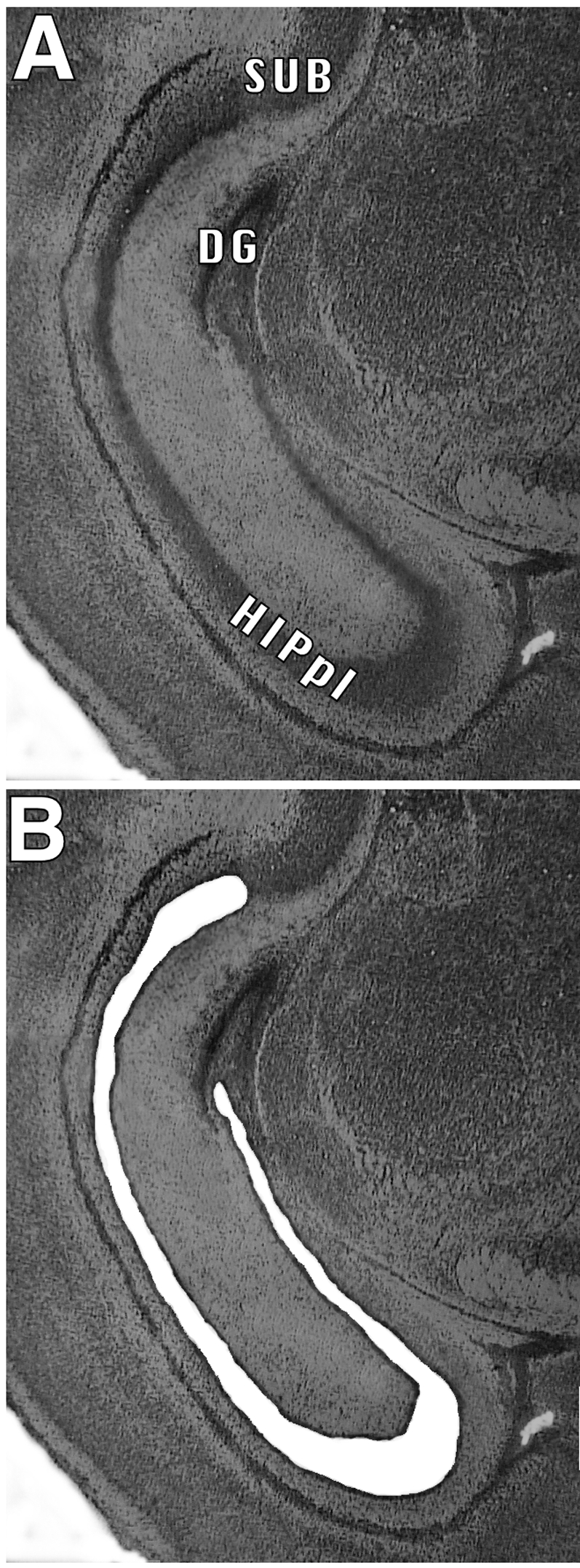
Histology of hippocampal sections before and after laser microdissection. Photomicrograph of a typical HistoGene-stained coronal section through the hippocampus of a 3-day-old mouse (a), and the same section following excision of the hippocampal pyramidal layer by microdissection on a Leica ASLMD laser capture microdissection system (b). HIPpl, hippocampal pyramidal layer; DG, dentate gyrus; SUB, subiculum. Note the precision of the cut.

Shown in Fig. 2 are micrographs of a section through the hippocampus before and after laser microdissection of the pyramidal layer. Examination of the section showed clean cut lines that adhered to the outline of the pyramidal layer with minimal tissue damage beyond the line of cut. The high quality of nucleic acids extracted from the dissected tissues, based on the NanoChip analysis results, further confirms the quality and integrity of the laser-excised tissues.

### Validation of the MeDIP Assay

**Figure 3:**
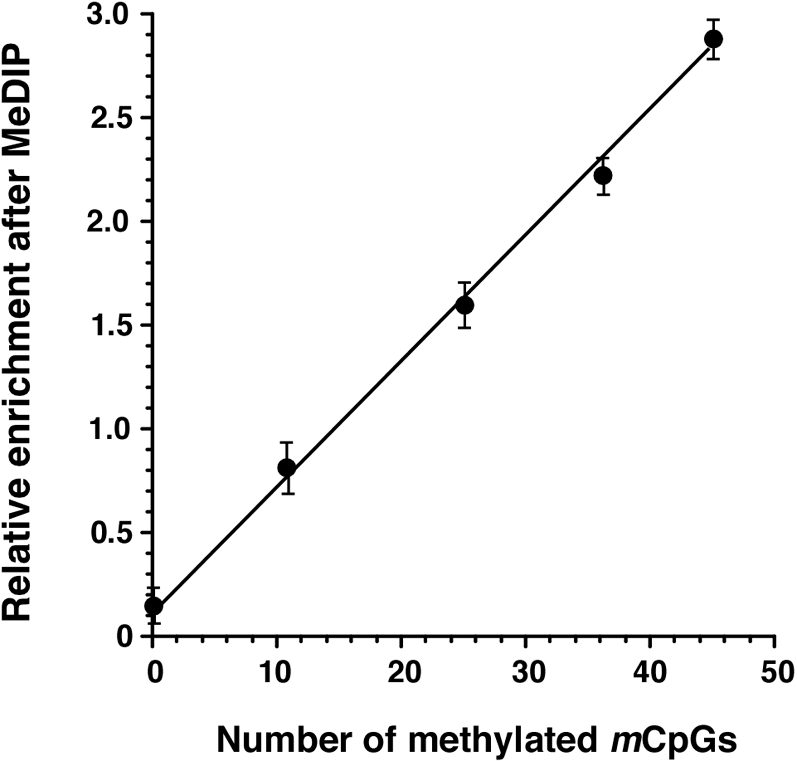
Relationship between the pixel density of MeDIP-generated signals and the number of methylated CpGs present in test DNA fragments of similar size (449, 446, 440, 445, and 449 bp) but containing different numbers of *methylated* CpGs (0, 11, 25, 36, or 45, respectively). Each data point represents the mean±SEM values for 3 clones. The linear regression line was fitted using GraphPad Prism software. Note the linearity of the plotted relationship, indicating that the MeDIP assay can quantitatively detect the number of methylated CpGs within a DNA fragment.

The ability of the MeDIP procedure to generate a signal that varies linearly with the number of methylated cytosines in a test DNA sample is crucial to the utility of the assay. To verify that the products of the MeDIP procedure are quantitatively proportional to the content of methylated cytosines in assayed samples, we tested four DNA fragments of similar lengths but containing different numbers of CpGs (see selected targets and corresponding primer sequences in Table 1). As shown in Fig. 3, densitometric values generated from the assay were directly proportional to the number of CpGs in the DNA fragments. This, thus, confirms the linearity of the MeDIP assay as applied in our laboratory for the present studies.

**Table 1:** PCR probes used in verification of the linearity of MeDIP-based methylcytosine detection.

Target	Forward (F:) and Reverse (R:) primers
446 bp fragment of UHNmmCPG0000038 CGI (contains 11 CPGs)	F: CAGGGTCTGTAGCAGGA
	R: TACCTCACGGCCCCAA
440 bp fragment of UHNmmcpg0007265 CGI (contains 25 CPGs)	F: ACCACTTTCCGTCCTCA
	R: CTTCTGCTGTCGCTGA
445 bp fragment of UHNmmcpg0000001 CGI (contains 36 CPGs)	F: GACCTAAATGTGGTGGA
	R: CTGGAGCGTTCCCGAA
449 bp fragment of UHNmmcpg0007250 CGI (contains 45 CPGs)	F: CGGAATCAGCGGGGAA
	R: CCCAAAAGGTCAGAAGGA

### Effects of Maternal Cocaine Administration on Global DNA Methylation and DNMT Expression in the Hippocampal Pyramidal Layer of P3 Pups

**Figure 4:**
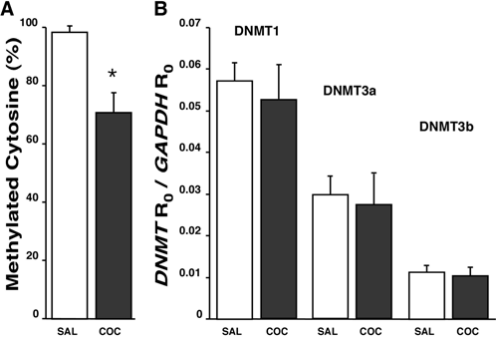
Global DNA methylation and DNMT expression levels in the hippocampal pyramidal layer of P3 offspring from saline-injected or cocaine-treated dams. (a) Global levels of cytosine methylation in DNA samples of control and cocaine groups each expressed as percentage relative to the methylation signal in Epigentek's methylated DNA standard. (b) Expression levels of DNA methyltransferases DNMT1, DNMT3a, and DNMT3b each calculated as a ratio relative to GAPDH as internal standard. Similar results were obtained with ß-actin used as internal standard. Each column represents mean±SEM of 10 samples. *p<0.05 compared to the saline-control group.

We compared global DNA cytosine methylation as well as DNMT1, DNMT3a, and DNMT3b expression levels in hippocampal pyramidal layers of male P3 pups born to cocaine-treated females compared with matched pups from saline-control dams. Shown in Table 2 are the sequences for the PCR primers and probes used for the analysis of the methyltransferases and the internal standards GAPDH and ß-actin. There was a significant decrease of approximately 30% in the global DNA methylation levels of cocaine-treatment P3 pups compared to matched saline controls (Fig. 4a). However, this decrease in global DNA methylation was not accompanied by changes in the expression levels of DNMT1, DNMT3a or DNMT3b (Fig. 4b). The lack of inter-group differences in DNA methyltransferase expression was independent of our use of either GAPDH or ß-actin as internal standards.

**Table 2:** PCR primers and probes used for analysis of DNA methylases and as internal controls.

Target	Forward (F:), reverse (R:) primers and probes (Pr:)
DNA methylase 1 (DNMT1)	F: GAGAACGGAACACACACTCTCACT
	R: TATTTGAGTCTGCCATTTCTGCTC
	Pr: *6FAM*-AAGCCAACGGTTGTCCCGCCA-TAMRA
DNA methylase 3a (DNMT3a)	F: CCATTCGACCTGGTGATTGG
	R: CGCGCATCATGCAGGA
	Pr: *6FAM*-TGCAATGACCTCTCCATTGTCAACCCT-TAMRA
DNA methylase 3b (DNMT3b)	F: AGCATCGTCTTCAGCAAGCA
	R: TATCCATACCCTCCTGATCTCCAT
	Pr: *6FAM*-TCATCCCCTGCCAGCGTCGAC-TAMRA
Glyceraldehyde-3-phosphate dehydrogenase (GAPDH)	F: CATGGCCTTCCGTGTTCCTA
	R: TGCTTCACCACCTTCTTGATGT
	Pr: *6FAM*-CCCCAATGTGTCCGTCGTGGATC-TAMRA
ß-actin	F: ACGGCCAGGTCATCACTATTG
	R: CAGGATTCCATACCCAAGAAGG
	Pr: *6FAM*-ACGAGCGGTTCCGATGCCCTG-TAMRA

### Profiles of CGI Methylation in Hippocampal Pyramidal Tissues of P3 Pups

**Figure 5:**
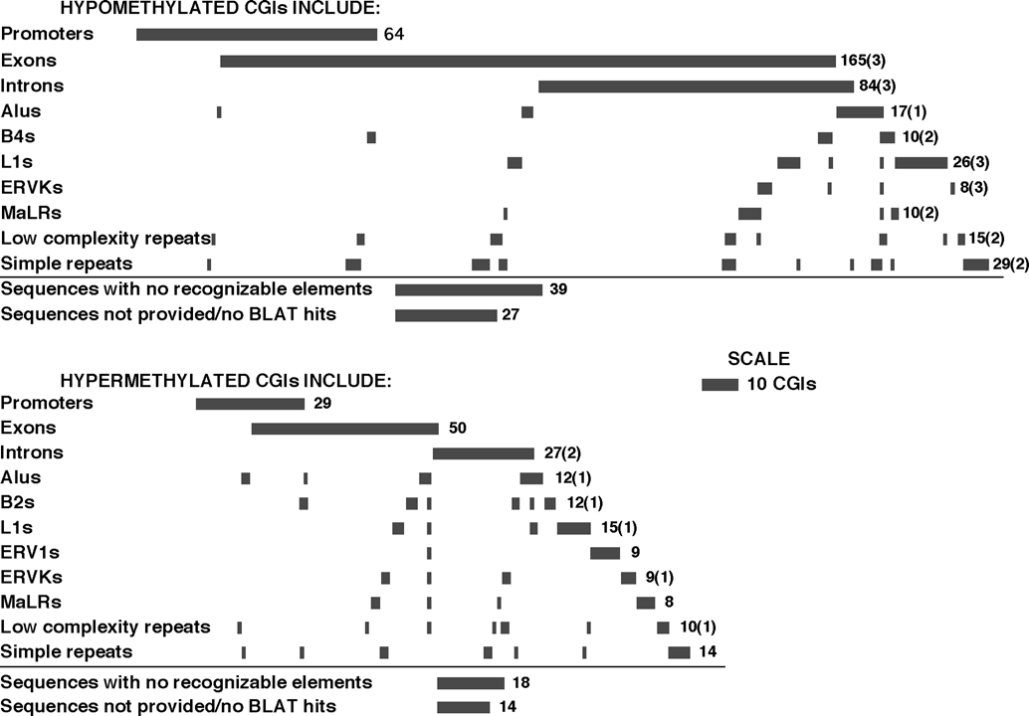
Genomic characteristics of abnormally methylated CGIs in the hippocampal pyramidal layer of P3 cocaine-treatment pups. Information associating abnormally methylated CGIs with various genomic elements was obtained by BLAT analysis of the affected CGIs as determined from the MeDIP/CGI array profiling experiments. The data is limited to the genomic elements that are present in at least 5 CGIs. The number of CGIs associated with a particular genomic element is represented by horizontal bars across from the element's name. The length of the bar is proportional to the number of CGIs; this number is also given at the end of the row. The number of CGIs with sequences matching more than one position in the genome is provided in parenthesis. The tendency for CGIs to be associated with more than one genomic element is reflected in the partial overlap of the bars on most rows. Note that overall there are more hypomethylated CGIs than hypermethylated CGIs.

MeDIP+CGI array-based analysis was undertaken to compare CGI methylation rates in hippocampal pyramidal tissues of male P3 pups from cocaine-treated pregnant females and their matched saline controls. Statistically significant differences in methylation were observed for 492 CpG islands (Fig. 5). Among the affected CGIs, 34% were hypermethylated (166 CGIs), while 66% were hypomethylated (327 CGIs) in the offspring of cocaine-exposed mothers. Further examination revealed that 93 CGIs having altered methylation states (19% of the affected CGIs) were associated with gene promoters (Fig. 4). Among these promoter-associated CGIs, 31% were hypermethylated and include CGIs associated with the promoters of 5 neural and 24 housekeeping genes; the 69% that were hypomethylated include CGIs associated with the promoters of 7 neural and 57 housekeeping genes. Exons were associated with 27% of the affected CGIs (134 CGIs); 37% of these CGIs were hypermethylated, while 63% were hypomethylated. Introns were associated with 9% of the CGIs with altered methylation states (44 CGIs); 61% of these were hypermethylated, and the remaining 39% were hypomethylated (Fig. 4). Repetitive elements, including members of the transposon families of LINE (long interspersed nuclear elements) such as L1, SINE (short interspersed nuclear elements) such as Alu, B2 and B4, and LTR (long-terminal repeat)-containing elements such as ERV1, ERVK, MaLR (described in [37]), as well as largely centromeric low complexity and simple repeats (described in [38]) were associated with 201 CGIs with altered methylation states in the offspring of cocaine-treated mice (41% of all affected CGIs). Among these CGIs, 36% were hypermethylated, and 64% were hypomethylated. The numbers of hypermethylated and hypomethylated CGIs containing specific frequently-encountered repetitive elements are given in Figure 5. For 98 of the CGIs with altered methylation states in the cocaine-treatment group (20% of total affected CGIs), however, we were unable to ascertain association with any recognizable genomic elements.

### Detailed CpG Methylation Analysis of Selected Promoters Associated with Hypermethylated or Hypomethylated CGIs and the Expression Levels of Genes Associated with these Promoters

**Figure 6:**
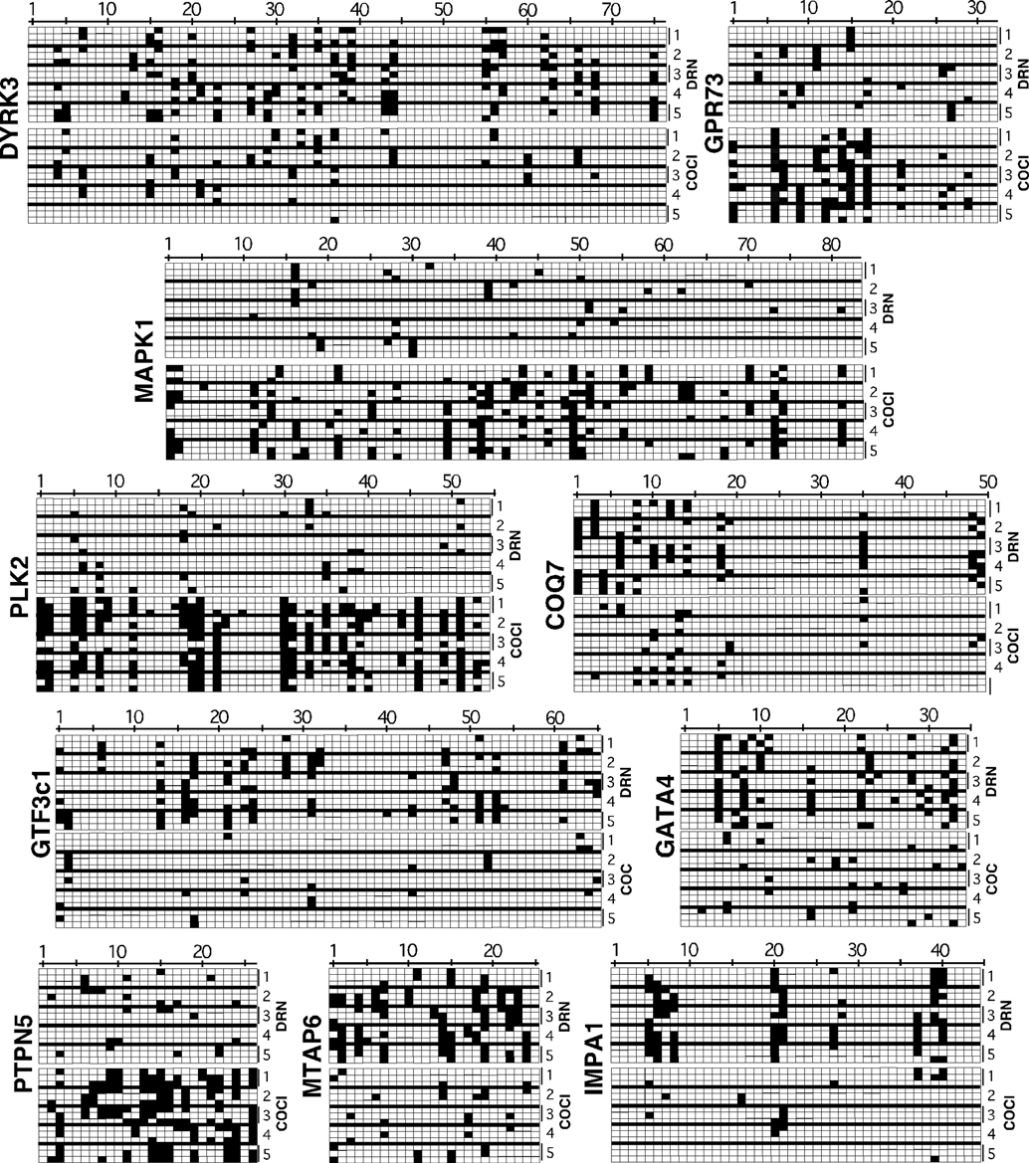
CpG methylograms of selected abnormally methylated promoters in P3 mice. From the MeDIP/CGI array-generated methylation profiles, ten cocaine-induced abnormally methylated CGIs known to be associated with gene promoter regions were selected and cloned, followed by bisulfite sequencing to identify the relative locations of methylated CpGs in the gene promoter sequence. For each chart, the name of the promoter is shown on the left vertical while the horizontal numbers mark the CpGs on the sequence; the CpGs are numbered from the 5'end of the clone. The vertical numbers on the right (1–5) indicate data rows for samples from each of 5 animals from the control (upper, DRN) and cocaine (lower, COC) groups. For each sample, three clones were sequenced, hence the three sub-rows available to each sample for methylation mapping. For each sub-row representing a clone, white squares indicate unmethylated CpGs while black-filled squares indicate methylated CpGs. If all three clones were consistent, the filled boxes would appear for each sub-row for that sample. If all 5 samples (mice) were consistent, then the filled boxes would extend vertically across all 5 rows. The relative density of filled boxes between the control (upper 5) samples and the cocaine (lower 5) samples provides a visual feedback on the relative levels of CpG methylation in these DNA regions. For example, clones of DYRK3 or COQ7 promoters contained lower proportions of methylated CpGs in the cocaine-treatment pups than in the saline-control offspring, whereas the converse applies in the case of GPR73 or MAPK1 clones.

This part of the study was aimed at verifying and further specifying the results of the foregoing MeDIP/CGI array-based methylation profiling analyses. Bisulfite sequencing analysis with evaluation of the expression levels of the linked genes was applied to 10 promoters selected from among those that were found from the high-throughput MeDIP/CGI profiling step to be associated with CGIs that displayed abnormal cocaine-induced methylation states. The identities of the genes associated with the selected promoters as well as the primers and probes used in bisulfite sequencing and in real-time PCR quantification of expressed mRNAs are shown in Table 3. First selected were the promoters for 4 genes that were potentially associated with hypermethylated CGIs in P3 offspring of cocaine-exposed mice. These included genes encoding GPR73 (G-protein coupled receptor 73), PLK2 (polo-like kinase 2), PTPN5 (protein-tyrosine phosphatase non-receptor type 5), and MAPK1(MAP Kinase 1). The promoters of the other 6 genes were likely associated with hypomethylated CGIs in P3 pups of cocaine-treated mice. These genes encode COQ7 (coenzyme Q7), DYRK3(dual-specificity tyrosine (Y)-phosphorylation-regulated kinase 3), GATA 4 (GATA binding protein 4), GTF3c1(subunit 1 of general transcription factor 3c), IMPA1 (inositol (myo)-1-monophosphatase), and MPAT6(microtubule-associated protein 6). Analysis of the methylograms derived from bisulfite sequencing suggested that methylated CpGs were present in the majority of the clones for all 10 promoters from samples of both study populations (Fig. 6).

**Table 3:** Primers and probes used in bisulfite sequencing and for real-time PCR.

Targets	Nested PCR primers used in bisulfite sequencing of CGI-associated promoters	Real-time PCR TaqMan primers and probes for mRNA quantification
G-protein coupled receptor 73 (GPR73; *PRK1*)	*1) F1-R1, 879 bp; 2) F2-R1, 790 bp.*	
	F1: TATTTGGAAATTTGGTTATGTTGTT	F: GGCATCGGCAACTTCATCTT
	R1: CCCTAATCTTTCCTAAAATTATTCTC	R: CGATAAGCAGGTTGGTGAGGTT
	F2: GGAAATTTGGTTATGTTGTTGAGTAG	Pr: *6FAM*-CACTGCCCTGGCCCGCTACAA-TAMRA
Polo-like kinase 2 (PLK2)	*1) F1-R1, 700bp; 2) F2-R1, 600bp.*	
	F1: GTTGTTTTGGTTTTGTTTGGTG	F: GCAAGAGTCCTTCGAGGATGTC
	R1: AACTACCCATCTTCAAAAAACTACTAC	R: CGAGAGCTGGTACCCAAAGC
	F2: ATGATAGGGTGTGGGGTTGTATATA	Pr: *6FAM*-TCCCCAAAGAGCAGCTGAGCACGT-TAMRA
Protein-tyrosine phosphatase non-receptor type 5 (PTPN5; *STEP*)	*1) F1-R1, 704bp; 2) F2-R1, 612bp.*	
	F1: GGGGTGAAATGTTAGGAATAAGTTT	F: AGTGCAGGGATTGGGAGAACT
	R1: AAACCCTTAACATAAAATCCCATCT	R: ACTGTTCGCATGTCTGGATCAT
	F2: GTTGGAATTATTTTTTTTAGTAGT	Pr: *6FAM*-CTGCTTCATTGCCACCAGCATCTGC-TAMRA
Mitogen activated protein kinase 1 (MAPK1)	*1) F1-R1, 855 bp; 2) F2-R1, 780 bp*.	F: CCACCCATACCTGGAGCAGTA
	F1: GGTTGGTGTGTAGTGAGTTTTTTG	F: CCACCCATACCTGGAGCAGTA
	R1: CCCCAAAACTCCAAAAATAAAA	R: TCGTCCAACT CCATGTCAAACT
	F2: AGTATTAGGATAGTGGGTGGGATTT	Pr: *6FAM-* TGAGCCCATTGCTGAAGCGCC-TAMRA
Coenzyme Q7 (COQ7)	*1) F1-R1, 857 bp; 2) F2-R1, 749 bp.*	
	F1: GATTTTAGTTGTTATATTAATGGATATGT	F: GGAGCTTGAACACCACGATACA
	R1: ACAAAACATCCTACAAAAATTCACC	R: CTGCATCCGGCCTGGATA
	F2: TTATGAATAATTTAGTAAAGGATGAATAA	Pr: *6FAM*-AGCTGGCTCCCGCGTATGCCTT-TAMRA
Dual-specificity tyrosine phosphorylation regulated kinase 3 (DYRK3)	*1) F1-R1, 800 bp; 2) F2-R1, 753 bp.*	
	F1: TTTGTAGTTTTAGGAAATTTGATGG	F: CCTGGACGCTCTGCACAA
	R1: ATCTCTACTACTCCACCCCAAAAC	R: GCTGGAGCCAAAGTCGATGA
	F2: TTTTTTTTATTTTTATTTTAATTGT	Pr: *6FAM*-ACACCACGGCCGAAGCGCC-TAMRA
GATA binding protein 4 (GATA4)	*1) F1-R1, 753 bp; 2) F2-R1, 636 bp.*	
	F1: GTTTTGTGATTGTTTTATTAATTTTTAGAT	F: CCCACTCTGGAGGCGAGAT
	R1: CCCAACAAACAAAATCCATAC	R: CCGGTTGATGCCGTTCAT
	F2: TTTATTTTTATTTTAGGGAAGGAATTATAG	Pr: *6FAM*-ACCTGTGCAATGCCTGTGGCCTC-TAMRA
General transcription factor IIIc subunit 1 (GTF3c1; *TFIIIc220*)	*1) F1-R1, 861 bp; 2) F2-R1, 858 bp.*	
	F1: ATTATTTTAAAGGATTGTTGTGAAA	F: GTTTTTGTGGCGGGCTTTAG
	R1: TTCCAAAAACAATAAAAAAACAAAC	R: AGTCGATTTCTTCATACCGGTCTT
	F2: ATTTTAAAGGATTGTTGTGAAA	Pr: *6FAM*-CACGCACCCGGGCATCAGTTTCTAC-TAMRA
Inositol (myo)-1-monophosphatase 1 (IMPA1)	*1) F1-R1, 707 bp; 2) F2-R1, 421 bp.*	
	F1: TGAGAGTGTTTAATGATAGTTATAATTTTA	F: GGAGGAGCAGATGCCTATTATGA
	R1: AAACCCTAAAAACTTAAACAAAACC	R: ACGGTCCACCCGTGACAT
	F2: GGATTAGTTTTTATAAAGGTAAATT	Pr: *6FAM*-TGATGCCAGCTCCCGCCATGT-TAMRA
Microtubule-associated protein 6 (MTAP6; STOP; MAP6)	*1) F1-R1, 757 bp; 2) F2-R1, 572 bp.*	
	F1: TTAGTATTTGGGAGGTAAAGGTAGG	F: CCCGACGACAAGGAGCAA
	R1: CAAAAAAACTATAAACTTAAACCATAAC	R: GGACCTCGGTTCTTAGATGCAT
	F2: TTTGAAATAGATATTTTATGGAG	Pr: *6FAM*-AGAGAGCCGGGTCAAACCCACCA-TAMRA

For the nested PCR, the first row for each gene depicts the primers used in the first and second steps of the nested PCR with the size of the expected amplicons. Common abbreviated names of the target genes are shown in parentheses. F1 and F2, forward primers; R1, reverse primer; Pr, probe.

Now, clones of promoters linked to COQ7, DYRK3, GATA4, GTF3c1, IMPA1, and MTAP6 from the pups of cocaine-treated mice contained clearly lower proportions of methylated CpGs than the parallel clones from the offspring of saline-control animals (Fig. 6 and Table 4). This inference is supported by the quantitative comparative analysis of the percentage of methylated CPGs in the clones for these promoters between the two treatment groups (6–14 fold, depending on the promoter). In contrast, visual observation as well as statistical evaluation of methylograms of the clones for GPR73, MAPK1, PLK2, and PTPN5-linked promoters demonstrated a significant increase in CpG methylation associated with cocaine exposure (4–12 fold, depending on the promoter; Fig. 6). These observations are in general agreement with the findings from the CGI methylation profiling experiment, which served as a basis for the selection of the promoters for the bisulfite sequencing analysis.

**Table 4:** Comparison of the MeDIP/CGI array profiling-defined methylation states of promoter-associated CGIs, promoter bisulfite sequencing-derived percentages of methylated CpGs, and RT-PCR-based expression levels for 10 selected genes between the hippocampal pyramidal layer of 3-day-old male offspring of saline-control mothers and the corresponding tissue of parallel pups born by cocaine-treated females.

Genes with CGI-containing promoters	CpG methylation of promoter-associated CGIs (MeDIP/CGI array profiling) log2 (cocaine/saline) ±SEM	CpG Methylation of promoter-associated CGIs (Bisulfite sequencing) log2 (cocaine/saline)±SEM	mRNA Expression of CGI-containing promoters (Real-time RT-PCR) log2 (cocaine/saline) ±SEM
GPR73	0.93±0.07*	1.79±0.30*	−1.66±0.43*
PLK2	1.23±0.10*	2.69±0.47*	−2.09±0.23*
PTPN5	1.03±0.03*	2.52±0.03*	−2.56±0.33*
MAPK1@	1.30±0.03*	2.39±0.03*	0.20±0.20
COQ7	−1.49±0.03*	−1.40±0.40*	0.27±0.33
DYRK3	−1.26±0.07*	−1.33±0.43*	1.43±0.20*
GATA4	−6.41±0.01*	−1.63±0.17*	2.96±0.37*
GTF3c1	−2.86±0.01*	−2.52±0.17*	1.73±0.27*
IMPA1	−2.33±0.01*	−2.13±0.30*	0.37±0.43
MTAP6@	0.93±0.07*	−1.89±0.03*	2.36±0.20*

@ Neural tissue-specific gene.

* p<0.05; comparing the corresponding cocaine and saline groups (each comprising ten liters).

To evaluate whether the observed differences in the levels of methylation in the bisulfite sequenced promoters would translate to differential levels of expression of the linked genes, we resorted to quantitative PCR analysis. This analysis showed that cocaine-induced hypermethylation corresponded to a significant decrease in the expression of GPR73, PLK2 and PTPN5 genes (5–13 fold, depending on the gene; Table 4), while cocaine-induced promoter hypomethylation corresponded to a significant increase in the expression of DYRK3, GATA4, GTF3c1 and MTAP6 genes (4–19 fold, depending on the gene; Table 4). Nevertheless, neither hypermethylation of the promoter for MAPK1 nor hypomethylation of the promoter for IMPA1 was associated with significant alterations in the expression of these genes (Table 4). Similar inferences were reached irrespective of whether the levels of genes of interest were normalized against GAPDH or ß-actin as internal standards.

### Comparison of DNA Methylation and Gene Expression Among P3 and P30 Offspring of Control and Cocaine-Treated Mothers

**Figure 7:**
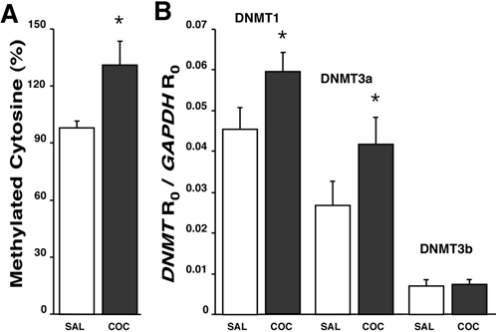
Effects of maternal cocaine exposure on global DNA methylation and DNMT gene expression in 30-day-old pups. (a) Relative levels of global DNA methylation in hippocampal pyramidal tissues of P30 pups. Each bar represents the mean±SEM (n = 10). *p<0.05 compared to saline-control group. (b) Expression levels of DNA methyltransferases relative to GAPDH as internal standard. *p<0.05 compared to the respective saline-control group.

We re-examined global DNA methylation, DNMT expression levels, and CGI methylation in hippocampal pyramidal tissues of 30-day-old brothers of the P3 mice used in generating the preceding data. In contrast to our findings in neonatal animals, we observed a significant elevation of approximately 35% in global DNA methylation in P30 offspring from cocaine-treated mothers compared to P30 offspring of saline-controls (Fig. 7a). Furthermore, this increase was accompanied by notably elevated expression levels of DNMT1 (by ∼30%) and DNMT3a (by ∼60%; Fig. 7b). No differences in the expression levels of DNMT3b were noted (Fig. 7b). DNMT expression data were similar whether analyzed with GAPDH or ß-actin as internal controls.

**Figure 8:**
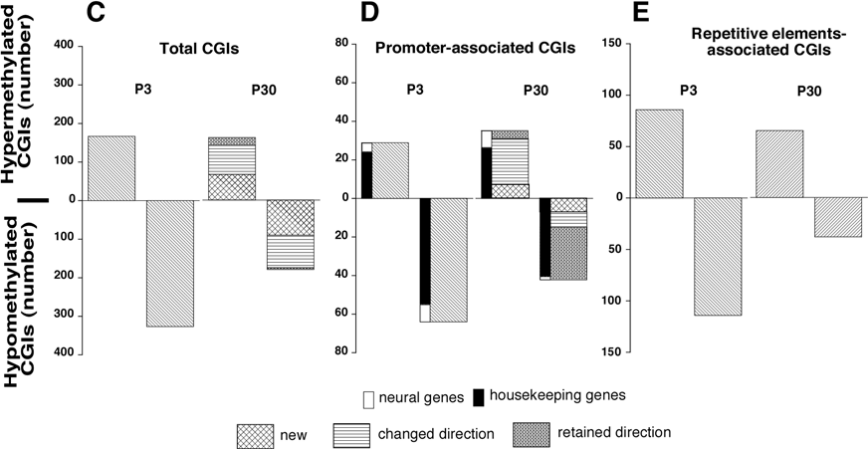
Effects of maternal cocaine exposure on CGI DNA methylation in P30 compared to P3 pups. (a) Cocaine-induced hypermethylated and hypomethylated CGIs in P30 compared to P3 pups. Shown are the numbers of CGIs with altered methylation levels in cocaine-treated animals relative to saline-controls at each age. For P30 data, the number of CGIs with altered methylation levels that did not show a change at P3 (*new*) are indicated by a cross-hatched segment of the bar. Also indicated by differential shading are CGIs that were significantly altered at both P3 and P30, retaining or reversing their direction of change at P30 compared to P3 (*retained direction; changed direction*). (b) Hypermethylated and hypomethylated promoter-associated CGIs at P3 and P30. For both ages, CGIs associated with promoters of neural tissue-specific genes (*neural genes*) are represented by white bars, while CGIs associated with promoters of non-neural tissue-specific genes (*housekeeping genes*) are represented by black bars. In addition, for P30, the number of CGIs with altered methylation state undetectable at P3 (*new*) and the number of CGIs corresponding to the CGIs affected at P3, which either retained or reversed the direction of change (*changed direction/retained direction*), are indicated as in a. (c) Abnormally methylated repetitive element-associated CGIs at P3 and P30.

MeDIP/CGI array-based profiling of hippocampal DNA samples of P30 mice detected altered methylation states for 278 CGIs in the cocaine-treated group compared to controls. This total number of altered CGIs is 42% fewer than the corresponding number in P3 animals (Fig. 8a). Among the CGIs with altered methylation states in P30 progeny of cocaine-treated mice, 59% (163 CGIs) were hypermethylated, and 41% (115 CGIs) were hypomethylated (Fig. 8a). Also, 67% of the CGIs (186 CGIs), which showed changes in methylation state in P30 cocaine group, corresponded to CGIs affected in P3 animals. However, 80 of these CGIs (43%) showed reversal in the direction of change: 4 previously hypermethylated CGIs became hypomethylated, and 76 previously hypomethylated CGIs became hypermethylated (Fig. 8a). In addition, 92 CGIs showed cocaine-related alterations in methylation state only in P30 animals (33% of the total number of the CGIs affected at this age). Among the newly altered CGIs, 73% were hypermethylated, while 27% were hypomethylated (Fig. 8a).

At P30, 77 CGIs with altered methylation state (28% of the total number of the affected CGIs) in the male offspring of cocaine-treated mice overlapped with gene promoters, which is 17% fewer promoter-associated CGIs than were detected at P3 (Fig. 8b). Among the promoter-associated CGIs in the DNA of P30 cocaine animals, 45% were hypermethylated (this includes CGIs associated with promoters of 9 neural and 26 housekeeping genes), and 55% (42) were hypomethylated (this includes CGIs associated with promoters of 2 neural and 40 housekeeping genes). Eighty-two percent of the promoter-associated CGIs showing altered methylation state at P30 (63 CGIs) were also present among the affected CGIs at P3. Among the latter CGIs, 39% (32 CGIs) reversed the direction of change: 8 previously hypermethylated CGIs became hypomethylated, while 24 previously hypomethylated CGIs became hypermethylated. At P30, 7 novel promoter-associated CGIs were hypermethylated, and 7 novel promoter-associated CGIs were hypomethylated. Further, 37% of the affected CGIs (103 CGIs) contained repetitive elements; 65 of those CGIs were hypermethylated (this is 49% fewer CGIs than at P3; Fig. 8c), while 38 were hypomethylated (this is 48% fewer CGIs than at P3; Fig. 8c). Thus, exposure of female mice to cocaine during pregnancy resulted in multiple alterations in the methylation states of offspring DNA, and some of these cocaine-related effects could persist through at least the prepubertal period of 30 days postnatum.

## Discussion

It is now widely believed that epigenetic phenomena contribute significantly to guiding mammalian development [7], [20], [39]–[42]. For instance, epigenetic mechanisms have been implicated in the programming of cell fate and differentiation [43]–[50], in defining ultimate activity levels of specific functional systems [22], [24], and in mediating environmental modulation of genetically-based developmental programs [22], [24], [42], [51], [52]. Indeed, the latter function probably subserves a unique biological role in enabling the formation of the phenotype that is best adapted for organismal survival and reproduction under the living conditions that are dictated by the developmental environment [52], [53]. Relatively easy environmental access to the developmental and reproductive machinery of a complex organism, however, could increase the organism's susceptibility to the adverse effects of abnormal prenatal and/or neonatal exposures to various chemical and biological toxicants [20], [23], [54]–[58]. This study demonstrates that maternal cocaine use could produce significant changes in the regulation of various offspring genes that may be involved in diverse functional systems. The study observed cocaine-related alterations in the expression of the DNA methyltransferases, DNMT1 and DNMT3b, in hippocampal pyramidal cells of pups from cocaine-exposed mothers. This observation is in agreement with, and extends, our earlier report on the ability of chronic cocaine exposure to affect the cellular levels of these two methyltransferases in the spermatocytes of adult male mice chronically exposed to cocaine [59]. Thus, parental exposure to cocaine, at least in the case of pregnant females, could have significant genomic and functional consequences upon the offspring.

The MeDIP procedure combined with CGI microarray technology enabled us to reliably and efficiently profile the methylation states of a wide range of CpG islands in multiple samples. By enriching the samples for CGIs prior to methylation analysis, we were able to focus on those regions of the DNA that are more likely to be associated with the regulatory sequences of various genes. Thus, many of the 492 CGIs that showed altered methylation in response to maternal cocaine exposure were found to be associated with the promoter regions of one or more genes. Among the ten altered CGIs that were selected for confirmation and detailed analysis by bisulfite sequencing, four were hypermethylated. These contained genes encoding G-protein-coupled receptor-73 which is a Gq-associated receptor for prokineticin that functions to support neuronal survival in the central nervous system [60], polo-like kinase2 which is known to promote cell proliferation and to participate in dendritic spine degradation [61], [62], protein-tyrosine phosphatase non-receptor type5 which is a neural tissue-specific dopamine D1/NMDA receptor-regulated tyrosine phosphatase involved in suppressing mitogen activated kinase cascades and blocking long-term potentiation [63], and MAP kinase1 which mediates extracellular signal-regulated intracellular cascades involved in cell differentiation and survival and in neuronal dendritic development and synaptic plasticity [64], [65]. The six genes whose promoter regions were found in hypomethylated CGIs include those encoding coenzyme Q7 which is a co-factor in mitochondrial respiration [66] and is essential for normal neurogenesis [67], dual specificity tyrosine (Y)-phosphorylation-regulated kinase3 which is a cyclic-AMP/protein kinase A-activated MAP kinase-related protein kinase implicated in regulating cell growth [68], GATA-binding protein4 which is a MAPK-activated zinc-finger-containing transcription factor involved in cell differentiation [69]–[72], subunit 1 of general transcription factor 3c which is a subunit of a polymerase III transcription factor complex [73], inositol (myo)-1-monophosphatase1 which dephosphorylates myo-inositol monophosphate in the phosphatidylinositol signaling system [74], and microtubule-associated protein 6, which is a neural-specific protein responsible for axonal microtubular stability [75]. This broad range of the targets of cocaine-induced alterations, and the crucial cellular functions of these transcripts, demonstrate the depth and breadth of the impact that chronic maternal cocaine exposure could exert on offspring neuronal function in the hippocampus and probably also in the cellular machinery of other tissues.

It is noteworthy that instances of both hypermethylation and hypomethylation were observed following maternal cocaine exposure. Generally, DNA CpG hypermethylation is thought to result in decreased expression of the affected genes, whereas hypomethylation could release the spatial constraints on access of the transcriptional machinery to the regulatory sites of the gene; this could thus enhance the chances of transcription of the gene [3]–[9]. By mediating both effects, we can expect that some genes would be activated while the expression of other genes would be repressed by chronic maternal cocaine exposure. Quantitative RT-PCR-based analysis of the expression levels of genes corresponding to selected promoters that we had found to be altered by cocaine exposure allowed us to test this notion in the present model. Indeed, cocaine-induced hypermethylation of several genes resulted in significant decrements of 5–15-fold in the expression of these genes, whereas cocaine-induced promoter hypomethylation caused significant increases of 4–19-fold in the expression of the corresponding genes. This notwithstanding, some genes, although showing altered methylation, did not demonstrate changes in expression. Hence, unlike in behavioral studies where cocaine is considered a psychostimulant, the genomic effects of chronic cocaine exposure are profound and multidirectional, but may not be simply predicted to be hypofunctional or hyperfunctional in impact.

In contrast to our findings in neonatal animals where cocaine exposure was associated with a decrease in global methylation levels, overall DNA methylation was significantly elevated in the P30 pups of cocaine-exposed mothers compared to matched controls. When we more closely examined the methylation patterns, various differences compared to the P3 animals were evident. Some P3 hypermethylated or hypomethylated CGIs remained as such, but newly hypermethylated or hypomethylated targets were also uncovered. Most intriguing were those CGIs that reversed the direction of response to cocaine at P30 compared to P3. Either they were hypomethylated at P3 and became hypermethylated at P30 or vice versa. One would have expected that with passage of time there might be extinction of the effects observed at P3, so that the DNA methylation states in the cocaine group would generally begin to approach the levels in the controls. At the present time it is not known if this reversal is an overcompensation, or if it reflects a different developmental trajectory initiated at birth or even *in utero*. Future studies could be designed to systematically probe these issues.

Cocaine remains one of the most abused substances in the Western hemisphere [76]–[80]. Maternal cocaine intake has been associated with diverse immediate and long-term deleterious effects on both the mother and the offspring [81]. While the specific consequences of cocaine use by pregnant women are still being debated [81]–[83], studies in several animal models have demonstrated significant deficits in multiple aspects of nervous system structure and function in the offspring of cocaine-exposed mothers (e.g.: [81], [84]–[86]. Considering that the epigenetic machinery is potentially vulnerable to abnormal chemical environments, it can be expected that one of the mechanisms by which cocaine exposure could produce neuroteratological effects might involve alterations in chromatin organization in neural cells. Such alterations in chromatin may in turn derive from abnormal states of DNA methylation induced by cocaine at various loci, including the CGIs.

The mechanism by which cocaine induces alterations in DNA methylation remains unknown. Classically, cocaine is thought to exert its biological effects by interfering with presynaptic reuptake of monoamine neurotransmitters from the synaptic cleft. The consequent dramatic increases in the synaptic concentrations of dopamine or other monoamines thus elicits the behavioral effects, including the motor, cardiovascular, and psychological effects [87]. Dopamine and other monoamines have been associated with teratological effects in animals [88]. Existing data appear insufficient to establish or discount a link between DNA methylation and the possible teratological effects of the catecholamines. In the same vein, it remains to be determined if maternal cocaine administration at these experimental doses is able to penetrate the placental barrier and alter fetal brain monoamine transmitter levels sufficiently to mediate these types of epigenetic alterations.

In summary, this study has demonstrated that maternal cocaine exposure during the second and third trimesters of gestation results in significant epigenomic alterations that are evident in the male offspring around the perinatal and prepubertal periods. The changes in hippocampal DNA methylation and gene expression were not accompanied by observable differences in gross tissue histology, overall quality of DNA, or even the litter sizes among the cocaine and control groups. Notwithstanding any overt structural differences, it is reasonable to speculate that the marked alterations in the genomic apparatus associated with maternal cocaine exposure could contribute to the disruption of neural and behavioral functions observed in previous animal and human cocaine studies.

## Materials and Methods

### Animals and cocaine treatment

Twenty timed pregnant CD1 dams (Charles River, Wilmington, MA) were maintained in individual cages in a climate-controlled room on a 12-h light/dark cycle. Ten randomly selected dams (the cocaine-treatment group) were subcutaneously injected (at the dorsum of the neck) with 20 mg/kg cocaine hydrochloride (Research Technology Branch, National Institute on Drug Abuse, Rockville, MD) dissolved in 200 µl of 0.9% saline, twice a day (at 8:00 AM and 8:00 PM) from the 8^th^ through the 19^th^ day of gestation (E8–E19). Animals in the second set (saline-control group) were subjected to the same schedule of injections but with 200 µl of 0.9% saline only. This model of prenatal cocaine exposure has been successfully used in multiple studies by our laboratory [89], [90] and by other groups [85], [91], [92]. The period of treatment was selected to start prior to the time of hippocampal neurogenesis and ended at a time after all hippocampal pyramidal neurons should have been generated and begun to assemble into the pyramidal layer [93], [94]. Throughout the treatment, all mice were weighed daily. The control and treatment animals were pair-fed such that the daily amount of food (Mouse Chow; Ralston Purina Co., Saint Louis, MO) provided to each control dam was matched to the amount consumed by the paired cocaine-treatment dam. Water was available *ad libitum*. As previously demonstrated, this feeding regimen resulted in similar weight gains from E8 to E19 in both the treatment and control groups [90]. Animals were allowed to deliver at term (∼gestational day 20). From the time of birth, each litter was fostered by an injection-naive surrogate dam (from the same batch as the experimental animals) that had given birth within the preceding 24–72 h. On day P3, two male pups from each litter were identified based on anogenital distance [85]. The selected pups were deeply anesthetized with halothane and following decapitation their brains were collected. In addition, pieces of liver were dissected out to be used to verify the sex of the animals by means of Mm01339632-s1 gene expression assay for Sry sex-determining region of the Y chromosome (Applied Biosystems, Foster City, CA). The assay confirmed that all the selected pups were males. The rest of the pups in each litter were allowed to survive until weaned (P21), after which the male pups (identified by their genitalia) from each litter were housed in groups of 2-3 per cage. At P30, one male per litter was deeply anesthetized with halothane and following decapitation the brains were collected. All procedures for animal housing and use in these experiments were reviewed and approved by the University of Maryland Animal Care and Use Committee and conformed to the NIH Guide for the care and use of experimental animals.

### Laser microdissection of hippocampal neurons

Brains collected from the pups were cut into left and right hemispheres and immediately transferred to a sheet of aluminum foil placed over dry ice. After 5 min, the frozen tissue was placed in cryogenic vials and stored in a freezer at −80°C for a period not exceeding 2 months before use. Each brain hemisphere was initially cut into serial 12-µm-thick coronal sections using a Zeiss cryostat (Carl Zeiss, Thornwood, NY). All sections containing the hippocampus were mounted on sterile PEN foil-covered slides (Leica Microsystems, Wetzlar, Germany), and the slides boxed while still inside the cryostat and then transferred to a −80°C freezer. Within 2 weeks of sectioning, slides were processed, in groups of up to 20 at a time, for staining and microdissection. The staining was conducted in an RNAse-free laminar flow hood and included the following 30-sec steps: 57% ethanol, distilled water, HistoGene stain (Arcturus, Mountain View, CA), distilled water, 75% ethanol, 95% ethanol, and 100% ethanol. After that, the sections were air-dried for 30–60 min. Laser microdissection was performed on a Leica ASLMD laser capture microdissection system (Leica Microsystems, Wetzlar, Germany). All dissections were executed by a single investigator who had no knowledge of the group assignment of the samples. On every section, the pyramidal layer, encompassing the C1, C2 and C3 hippocampal regions, was identified based on Paxinos and Franklin [95] and carefully cut out.

For the P3 animals, tissues obtained from the left hemispheres of the two pups taken from each litter (20 male pups altogether) were combined and used for investigating CGI methylation. Pyramidal layer tissue from the right hemisphere of one of these brains was used for analyzing gene expression. Tissues excised from every 5^th^ section of the right hemisphere of the second brain were used for estimation of total DNA content. The remainder of the laser-excised hippocampal pyramidal tissue from these P3 pups was used for evaluating global DNA methylation.

For the P30 animals, given that a single brain was selected per litter, the laser-excised hippocampal pyramidal tissues from the left hemisphere were pooled and used for assessing CGI methylation. Pyramidal layer tissue cut out from every 10^th^ section of the right hemisphere was used for measuring total DNA content, while tissue from every 11th section was used for investigating gene expression. The remainder of the excised hippocampal tissue from the right hemisphere was used for determining global DNA methylation.

During each run, we captured and saved 10X and 40X microscope images before and after the laser microdissection of each brain section from which the tissues for total DNA assay were obtained. These images were later visually examined by two histologists in order to assess potential qualitative histological differences between cocaine-treatment and saline-control tissues and to verify the precision of the laser cut.

### Measurement of total DNA content in hippocampal pyramidal tissue

In estimating the DNA content of the hippocampal pyramidal layer of a hemisphere, we used the Cavalieri estimator as modified by Reed and colleagues [96]. As described above, the 12-µm sections used for DNA content estimation were selected by systematic random sampling (every 5^th^ section in P3 mice and every 10^th^ section in P30 animals). The amount of DNA in the excised pyramidal layer from each section was assayed using PicoGreen dsDNA quantitation kit (Invitrogen, Carlsbad, CA) and the resulting fluorescence detected on a SuperMax Gemini XS Spectrofluorometer (Molecular Devices, Sunnyvale, CA) at the emission wavelength of 520 nm and 480 nm for excitation. Salmon sperm DNA dilutions of known concentration served as standards. Based on the obtained value (*a_i_*) from each examined section, the total DNA content of the hippocampal pyramidal layer in a hemisphere (W_DNA_) was calculated as follows:
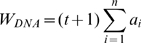
where *n* = number of sections examined, and *t* = number of non-assayed sections occurring between the sections examined. The precision of the data obtained for each animal was calculated as the coefficient of error (CE) for the modified Cavalieri method [96]. Statistical analyses included two-way ANOVA with maternal treatment and age of the offspring as independent variables. The ANOVA was followed by Tukey *post hoc* tests (Prism, GraphPad, San Diego, CA). Significant differences were inferred at *p* <0.05 or better.

### Global DNA Methylation Assay

Tissue DNA was extracted using Puregene DNA isolation kit (Gentra Systems, Minneapolis, MN) with glycogen added as a carrier to assist in DNA precipitation. The extracted DNA was quantified by PicoGreen dsDNA quantitation kit as described above. For each sample, methylation analysis was performed in triplicate aliquots (200 ng DNA each) using anti-methylated cytosine antibody-based MethylAmp Global DNA methylation quantification kit (Epigentek, New York, NY). We used methylated DNA standards supplied with the kit, while for blanks we simply omitted DNA from the assay. The spectroscopic end point (optical density, OD) was read on an Opsys MR microplate reader (Dynex, Chantilly, VA). After subtracting blank readings from the readings for both the sample and the standard, the value of DNA methylation for each sample was calculated as a ratio of sample OD relative to the OD of the standard. The triplicate values obtained for each tissue from each animal were averaged to give a single data point for the tissue/animal. Statistical analysis was conducted as described above for tissue DNA content.

### High-Throughput CGI Methylation Analysis

Methylation profiling analysis was performed using a modification of the MeDIP (methylated DNA immunoprecipitation) method of Weber and colleagues [97] combined with CGI microarray hybridization. MeDIP was selected because the methodology supports high-resolution and high-throughput DNA methylation profiling, is sensitive to the overall number of methylated cytosine residues in the regions of interest, and its sensitivity is independent of the presence of recognition sites for methylation-sensitive restrictases [97], [98]. DNA was extracted as described above for global methylation assay. The quantity and quality of the extracted DNA was determined by NanoChip on Agilent 2100 Bioanalyzer (Agilent Technologies, Palo Alto, CA). For all the samples, DNA fragments sized 100 kb or less constituted <87% of the total sample DNA, indicating that the quality of the DNA was appropriate for CGI methylation analysis.

For the MeDIP procedure, pairs of 400 ng DNA samples from cocaine-treated and saline-control tissues were incubated in parallel with MseI (T/TAA) restrictase (New England Biolabs, Ipswich, MA; 1 *U*, 37°C, overnight), which digests the bulk DNA into smaller fragments but retains CGIs largely intact [29]. Upon completion of the digestion, the DNA was purified with DNA MinElute reaction cleanup kit (Quiagen, Valencia, CA) and denatured for 10 min at 95°C. The denatured DNA was incubated for 2 h at 4°C with 0.1 µl of anti-methylated cytosine mouse monoclonal antibodies (Epigentek, New York, NY) in a final volume of 500 µl immunoprecipitation (IP) buffer (composition: 10 mM sodium phosphate, 140 mM NaCl, 0.05% triton X100, pH 7.4). The mixture was further incubated for 2 h at 4°C in 30 ml of sheep anti-mouse IgG M280 Dynabeads (Invitrogen, Carlsbad, CA). After washing three times with IP buffer, the beads were treated with proteinase K (2 *U*; Invitrogen, Carlsbad, CA) for 3 h at 50°C. Methylated DNA was then recovered by use of the MinElute reaction cleanup kit. The recovered DNA was processed for random-primed Klenow amplification with Bioprime kit (Invitrogen, Carlsbad, CA) in the presence of unmodified dNTPs for 5–10 fold sensitivity improvement. After another MinElute cleanup step, the DNA was labeled using 3DNA Array 900DNA detection system (Genisphere, Hatfield, PA). Briefly, the DNA fragments were tailed with dTTP and ligated with capture oligos complementary to either Alexa 564-incorporating dendrimers (for the saline-control samples) or Alexa 647-incorporating dendrimers (for the cocaine-treatment samples). Pairs of these tagged saline-control and cocaine-treatment samples were co-hybridized to a glass CGI microarray containing 7,296 annotated clones (Microarray Center of the University Health Network, Toronto, ON, Canada). For each array hybridization, the paired DNA samples used were matched between pups of litters generated by pair-fed saline-control and cocaine-treatment dams. Array hybridization was carried out overnight at 65°C, after which the arrays were incubated for 3 h at 65°C with a capture-reagent mixture containing both Alexa 546-incorporating dendrimers and Alexa 647-incorporating dendrimers. The arrays were washed and spin-dried in a CentraCL3 centrifuge (Thermo Electron, Wyman, MA). For both P3 and P30 assays, DNA from each litter was used once for hybridization, hence a total of ten P3 arrays and ten P30 arrays were processed. The microarray data are available in MethDB database (www.metdb.de): Sample ID21455-Experiment ID21524 (for P3 data) and Sample ID21456-Experiment ID21525 (for P30 data).

Microarray slides were scanned at 10-µm resolution on a GenePix 4100A scanner (Axon, Union City, CA), with the laser excitation set at 532 nm (green emission filter 575DF35; photomultiplier voltage 550) for the Alexa 546 (saline-control) samples, while the laser excitation was switched to 635 nm (red emission filter 670DF40; photomultiplier voltage 695) to obtain readings for the Alexa 637 (cocaine-treatment) samples. The signals were converted into high (16-bits-per-pixel) resolution images, providing a color depth of 65,536 levels. Densitometric quantitation was then conducted on the images using GenePix Pro 6.0 software (Axon, Union City, CA) with background subtraction. Quality control utilized the 384 positive and 24 negative QC spots present on the arrays. Intra-slide and inter-slide normalizations (the latter being for slides hybridized with samples of the same pup age) were performed by LOWESS transformation in Acuity 4.0 software (Axon, Union City, CA). Statistical analysis of the normalized data was conducted based on the Significance Analysis of Microarray (SAM) algorithm translated into Excel macros (http://www-stat.stanford.edu/tibs/SAM/). In the analysis, a threshold was set to identify the maximum number of altered CGIs at the minimal false discovery rate (FDR) indicated by SAM for a given set of microarray data. For analysis of both P3 and P30 samples, the FDR rates were <5%. CGIs revealing significantly altered methylation levels were subjected to a BLAT search (http://genome.ucsc.edu/cgi-bin/hgBlat) to identify their possible affiliation with specific promoters, exons, introns, and/or repetitive elements. The latter were recognized by the RepeatMasker algorithm (http://www.repeatmasker.org). The neural tissue-specific genes among the genes that were identified as linked to promoters associated with abnormally methylated CGIs were detected with PathwayAssist 3.0 software (Ariadne Genomics, Rockville, MD) based on the Entrez Gene database (http://www.ncbi.nlm.nih.gov/entrez/query.fcgiCMDDisplayDBgene) using the keywords: neural, neuron, glia, synapse, axon, and dendrite.

To verify the linearity of MeDIP-based methylated DNA detection, a source sample of hippocampal DNA was extracted from a drug-naive adult mouse. Using 4 sets of primers designed for the purpose, 4 amplicons were generated from the source DNA to yield fragments that were close in length but containing different numbers of CpGs. These fragments, named according to the CpGmouse database (http://data.microarrays.ca/cpgmouse), and their respective primers designed with GeneFisher (http://bibiserv.techfak.uni-bielefeld.de/genefisher), are presented in Table 1. The primers were synthesized by Applied Biosystems (Foster City, CA). PCR amplification of the fragments was conducted on a Sprint Thermal Cycler (Thermo Electron, Wyman, MA) employing PCR MasterMix (Fermentas, Hanover, MD). Cycle conditions (using PCR MasterMix) providing linear and compatible amplification of the selected fragments were predetermined to be: 94°C for 3 min; 94 cycles at 94°C for 30 sec; 52°C for Fr1, Fr2, and Fr3 and 48°C for Fr4 for 30 sec, and then for all 72°C for 30 sec. Two hundred nanogram of DNA was used for amplification of each fragment in 100 µl total volume. The reaction mixture with each amplicon was electrophoresed on ethidium bromide-stained 1.5% agarose gel followed by extraction of the PCR product with QIAquick gel extraction kit (Qiagen, Valencia, CA) and cloning these products using TOPO TA Cloning kit (Invitrogen, Carlsbad, CA). Selected colonies were checked for inserts by colony PCR with T7 and Sp6 primers (Fermentas, Hanover, MD). After transformation, the cells were grown on FastMedia LB-agar (Fermentas, Hanover, MD) overnight at 37°C. For each fragment, 3 colonies were then grown in 3 ml of FastMedia LB-liquid (Fermentas, Hanover, MD) with agitation overnight at 37°C. Plasmid DNA was extracted with Wizard plasmid DNA purification system (Promega, Madison, WI). Sequencing of the extracted plasmids by Macrogen (Seoul, South Korea) confirmed the predicted sequences of the DNA fragments. Afterwards, each clone was amplified by PCR as described earlier for the amplification of same fragments from the original DNA. The PCR products were cleaned by QIAquick extraction, and 2 µg of each product was treated with SssI methylase (New England Biolabs, Ipswitch, MA; 2 *U* for 1 h at 37°C) to methylate all CpGs present in the selected DNA sequences. Methylated DNA was cleaned using Qiagen's MiniElute, and 400 ng DNA from each methylated clone, together with 400 ng of unmodified DNA from Fr4 clones (selected because Fr4 contained the highest number of CpGs), was subjected to the MeDIP procedure. Following purification with Dynabeads, the recovered DNA samples were used for PCR-based amplification under the same conditions as described above (50 µl total volume). One microliter from each of the amplified samples was run on an ethidium bromide-stained 1.5% agarose gel. After electrophoresis, images of the gels were digitized under UV light using a LAS 3000 Image Analyzer (Fuji, Edison, NJ), and the densitometric analysis of the bands produced by the fragments of interest was performed using Science Lab 2001 software (Fuji, Edison, NJ). A linear regression fit of the data was achieved with KaleidaGraph Software (Synergy, Reading, PA).

### Bisulfite Sequencing

A detailed analysis of CpG methylation by bisulfite sequencing was performed for ten selected promoters associated with CGIs for which MeDIP/CGI array profiling had indicated significant cocaine-induced alterations in their methylation state (hypomethylation or hypermethylation at age P3). The promoter sequences were obtained from the Transcriptional Regulatory Element Database (http://rulai.cshl.edu/cgi-bin/TRED/tred.cgiprocesshome). For each of the ten P3 saline-control and cocaine-treatment samples, 400 ng DNA was subjected to sodium bisulfite treatment using EZ DNA Methylation-Gold kit (Zymo Research, Orange CA). The C-to-T conversion was conducted for 10 min at 98°C and then for 2.5 h at 64°C in a Sprint thermal cycler (Thermo Electron, Wyman, MA). The converted DNA was cleaned using Zymo-Spin IC columns, and then subjected to nested PCR amplification. Primers for each promoter (Table 3) were designed using MethPrimer (http://urogene.org/methprimer/index1.html) and synthesized by Applied Biosystems. The amplification procedure was performed in a total volume of 50 µl employing PCR MasterMix (Fermentas). For both the first and second steps of the nested PCR, the conditions were: 94°C for 2 min, 30 cycles at 94°C for 30 sec followed by 55–62°C (depending on the primers) for 1 min, and 72°C for 1 min. The PCR products were electrophoresed on ethidium bromide-stained 1.5% agarose gels, and extracted using Qiagen's QIAquick kit. Extracted DNA fragments were cloned and 3 clones of each fragment of interest for each sample DNA were collected as described above. For each clone, the plasmid DNA was extracted with Wizard plasmid DNA purification system (Promega) and sequenced by Macrogen (Seoul, South Korea). Detection of methylated cytosine in cloned sequences (3 clones per sequence per sample) was performed using BiQ Analyzer software (http://bioinf.mpi-sb.mpg.de/projects/biq-analyzer/index.html). The percentage of methylated CpGs in each cloned sequence was calculated, with the values from parallel sequences generated from the same DNA sample averaged to generate a single data point. Statistical analysis of the data was performed by GeneSpring software (Agilent, Palo Alto, CA) using two-tailed *t*-tests with the FDR multiple testing error correction set at the *p* cut-off value of 0.05.

To verify that there were no methylation-associated biases during the PCR amplification procedures we screened each set of nested primers according to the technique of Warnecke and colleagues [99] with slight modifications. Essentially, we evaluated the methylation levels of sequences obtained from bisulfite conversion of the tested primers using serial mixtures of DNA samples obtained from hippocampal tissues of two adult male mice, one from a saline-control litter and the other from a cocaine-treatment litter. The mixing started with 0% and ended with 100% of the DNA from the saline-control animal. For all the primer sets, the increase in the recovered methylated CpGs was linear (data not shown), indicating the absence of significant bias among the samples.

### Quantitative Real-Time RT-PCR

Real-time PCR was conducted to analyze the expression of DNMTs in P3 and P30 tissues, and also to evaluate the expression of genes associated with the promoters examined by bisulfite sequencing as described above. RNA was extracted from designated tissues by RNeasy Micro kit (Qiagen, Valencia CA). To remove genomic DNA contamination, the isolated RNA was treated with amplification grade DNAse I (1 *U*/ µg RNA; Invitrogen, Carlsbad, CA) for 15 min at 25°C. The quantity and quality of the obtained DNA were determined by NanoChip on an Agilent 2100 Bioanalyzer. All RNA samples used showed a 28S to 18S ratio >1.5 and the absence of low molecular weight smears. Reverse transcription was performed with Qiagen's Omniscript RT kit using random hexamers as primers (20 µl of RT reaction reagent/1 µg RNA). Real-time PCR utilized TaqMan approach with glyceraldehyde-3-phosphate dehydrogenase (GAPDH) or ß-actin employed as endogenous controls. The TaqMan PCR primers and probes (Table 3) were designed using Primer Express 1.5a (Applied Biosystems) and synthesized by Applied Biosystems. The PCR reactions were carried out on an ABI Prism 7700 Sequence Detector (Applied Biosystems). All PCR reactions were run in a monoplex mode–amplification of one gene per reaction well in a PCR plate. Each reaction included 1 µl cDNA in a total volume of 50 µl and used TaqMan Universal Master Mix (Applied Biosystems). Cycling parameters were: 95°C for 10 min, followed by 45 cycles of 95°C for 15 sec and 60°C for 1 min. Assays were performed in triplicate, and the values averaged to obtain one datum per sample. Procedures for normalization to internal standards and for expression of the resultant data were according to the amplification plot method of Peirson and colleagues [100] as implemented in DART-PCR Excel workbook (http://www.gene-quantification.info/bestkeeper.html). Amplification efficiencies for both target and control genes were calculated from the raw data around the midpoint of the transformed signal range, an approach that is thought to be more accurate than if derived from an external standard curve [101]. Our “standard cDNA” used for efficiency calculations was generated from a “standard” hippocampal tissue obtained from a drug-naive adult male mouse. To assess possible contamination with chromosomal DNA, several RNA samples were processed for PCR with GAPDH probe and primers without the RT step. In each case, no amplification products were detected. Gene expression data obtained with use of GAPDH or ß-actin as internal standards were separately analyzed with similar conclusions reached. Multiple parallel t-tests were conducted to compare corresponding P3 and P30 data. All comparisons were based on 10 saline-treated and 10 cocaine-treated samples, with statistical significance inferred at *p*<0.05 or better.
